# Tentacle-shaped mesh for fixation-free repair of umbilical hernias

**DOI:** 10.1007/s10029-019-01950-8

**Published:** 2019-04-12

**Authors:** G. Amato, G. Romano, A. Agrusa, G. L. Canu, E. Gulotta, E. Erdas, P. G. Calò

**Affiliations:** 1 Postgraduate School of General Surgery, University of Cagliari, Cittadella Universitaria di Monserrato, 09042 Monserrato - Cagliari, Italy; 2 Department of General Surgery and Urgency, University of Palermo, Palermo, Italy; 3 Department of General Surgery, University of Cagliari, Cagliari, Italy

**Keywords:** Hernia, Umbilical, Hernia repair, Tentacle mesh, Fixation free, Friction force, Freexation

## Abstract

**Purpose:**

Mesh fixation and broad overlap represent an open issue in umbilical hernia repair. A proprietary-designed implant with tentacle straps at its boundary has been developed to ensure a suture-free repair and a broader coverage of the abdominal wall. The study describes the results of umbilical hernia procedures carried out with the tentacle-shaped implant and the related surgical technique.

**Methods:**

A proprietary tentacle-shaped flat mesh having a central body with integrated radiating arms at its edge was used to repair large umbilical hernias in 62 patients. The implant was placed in preperitoneal sublay. The friction of the straps, crossing the abdominal wall thanks to a special needle passer, was intended to assure adequate grip to hold the implant in place assuring a fixation-free procedure and broad overlap of the hernia defect.

**Results:**

In a mean follow-up of 48 months (range 10–62 months), 4 seromas and 2 ischemia of the navel skin occurred. No infections, hematomas, chronic pain, mesh dislocation, or recurrence has been reported.

**Conclusions:**

The tentacle strap system of the prosthesis effectively ensured an easier implant placement avoiding the need for suturing the mesh. The arms of the implant ensured a proper orientation and stabilization of the mesh in association with a broad defect overlap. The specifically developed surgical procedure showed a quick postoperative recovery, a very low complication rate, and no recurrences even in the long term.

## Introduction

Umbilical hernia is a rather frequent pathology, having an incidence of 10–14% compared to all hernias. For decades, it was performed with direct suture technique with recurrences up to 54% [1, 2, 3]. In recent years, the use of synthetic implants has contributed to a drastic reduction in recurrences [4, 5, 6]. Nevertheless, prosthetic umbilical hernia repair is affected by frequent, specific postoperative complications consequent to mesh fixation and insufficient overlap of the mesh over the hernia opening, which can lead to tissue tearing, mesh migration, and recurrences [7]. To eliminate these problems, a prosthetic device has been developed, composed of an oval flat mesh with incorporated tentacle straps at its boundary, that allows a simple, fast "fixation free" positioning of the implant, assuring broad coverage of the umbilical region. This implant is delivered by mean of a proprietary passer that allows simple, quick deployment of the device distant from the hernia edge. The mesh is held in place without fixation by the tentacle straps that are pulled out from the preperitoneal space to the subcutaneous space passing through the rectus muscles and fascia. The present report aims to demonstrate the effectiveness of the use of an adequately sized tentacle-shaped mesh in patients suffering from umbilical hernia defects larger than 2 cm in diameter.

## Materials and methods

**Fig. 1.**
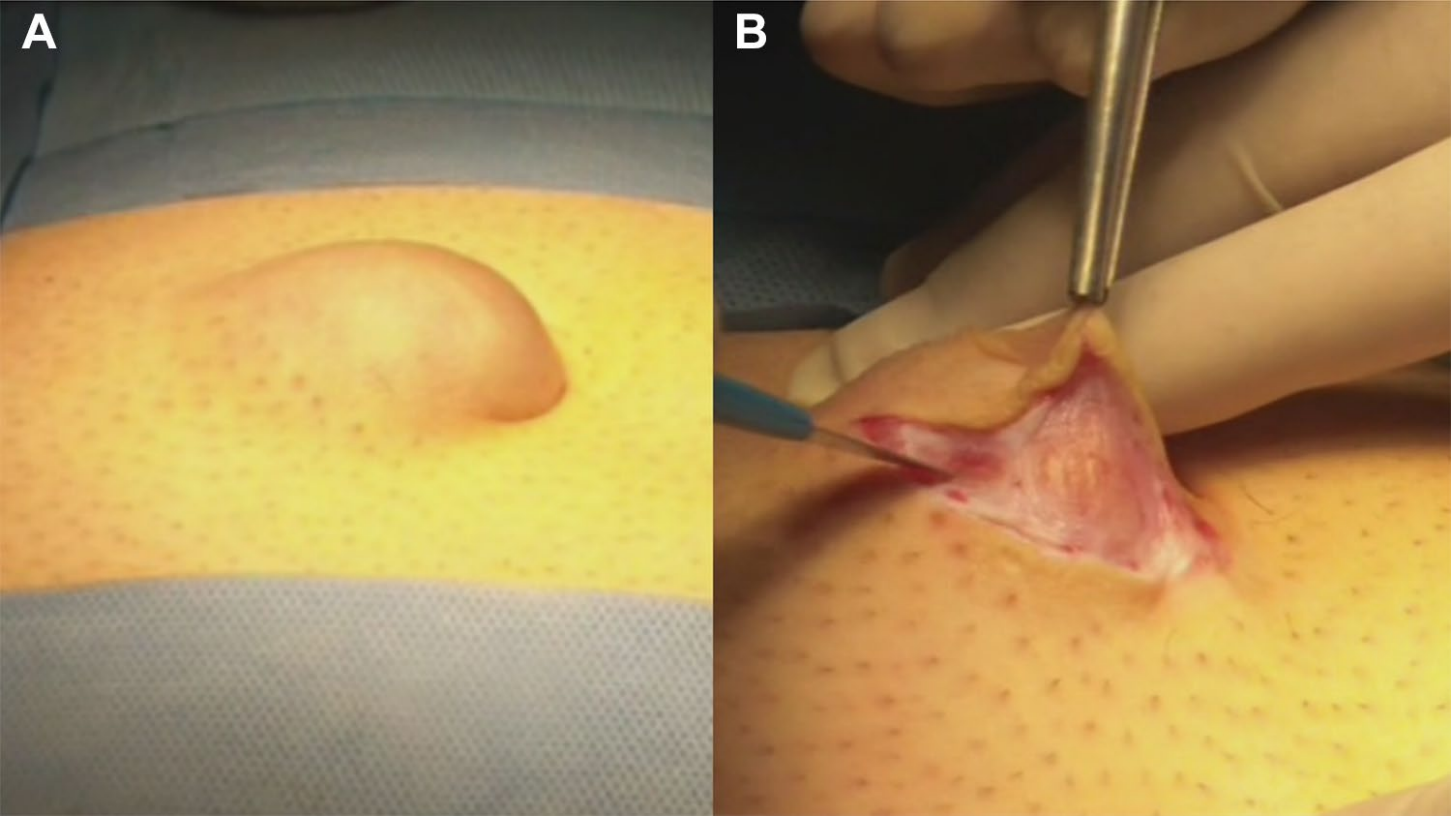
**a** Preoperative aspect of large incarcerated umbilical hernia. ** b** Skin incision at the edge of the umbilical hollow

**Fig. 2.**
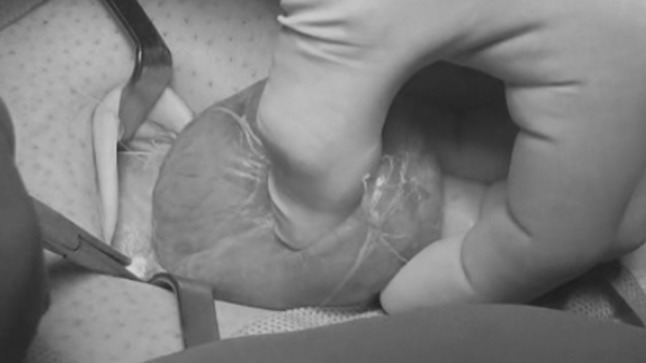
The hernia content is clearly incarcerated into the sac. Stump dissection of the adhesions between hernia sac and linea alba

**Fig. 3.**
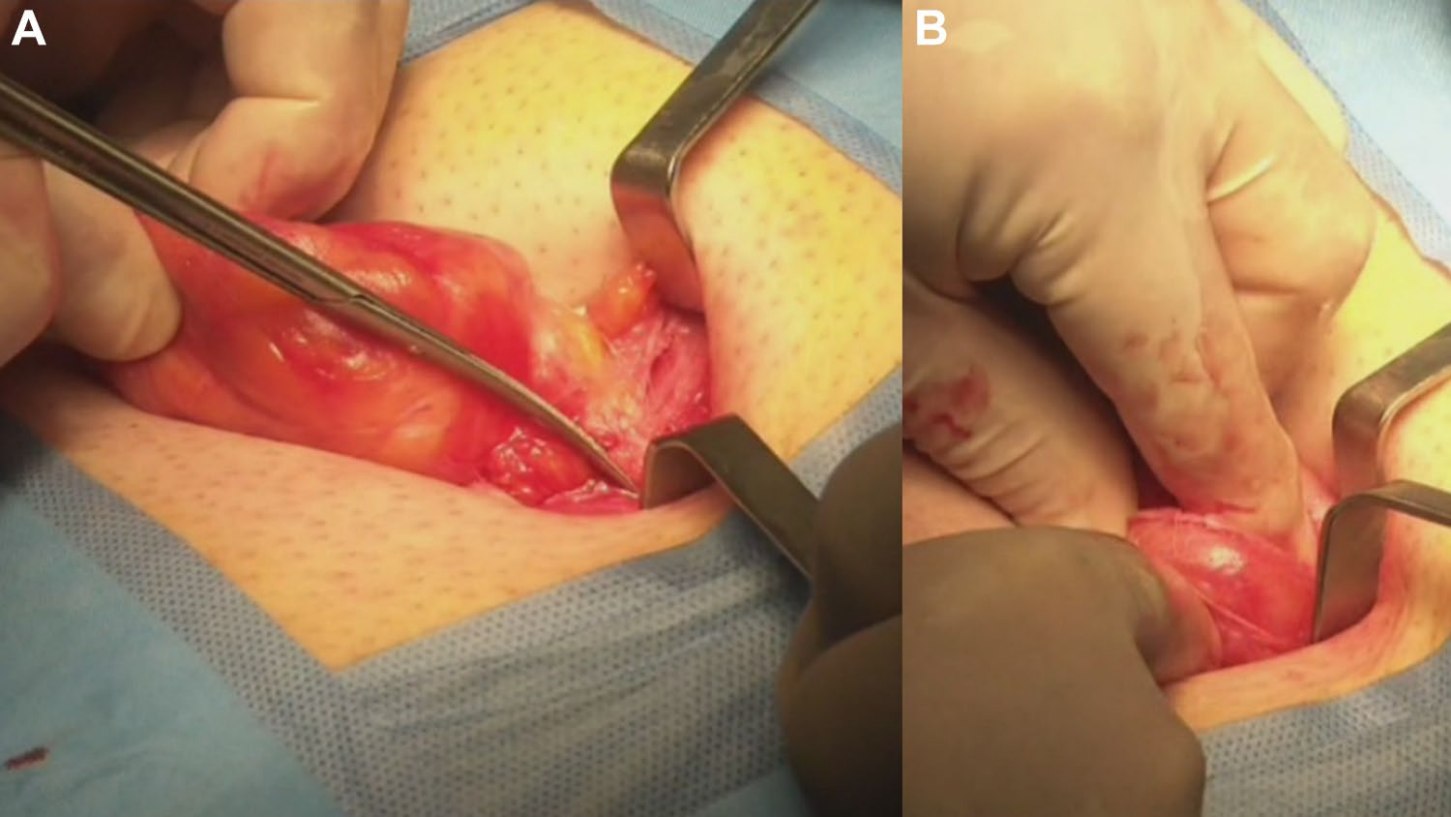
**a** After adhesiolysis, incision of the linea alba to release the incarcerated hernia content.** b** The hernia content is being reduced into the abdominal cavity

**Fig. 4.**
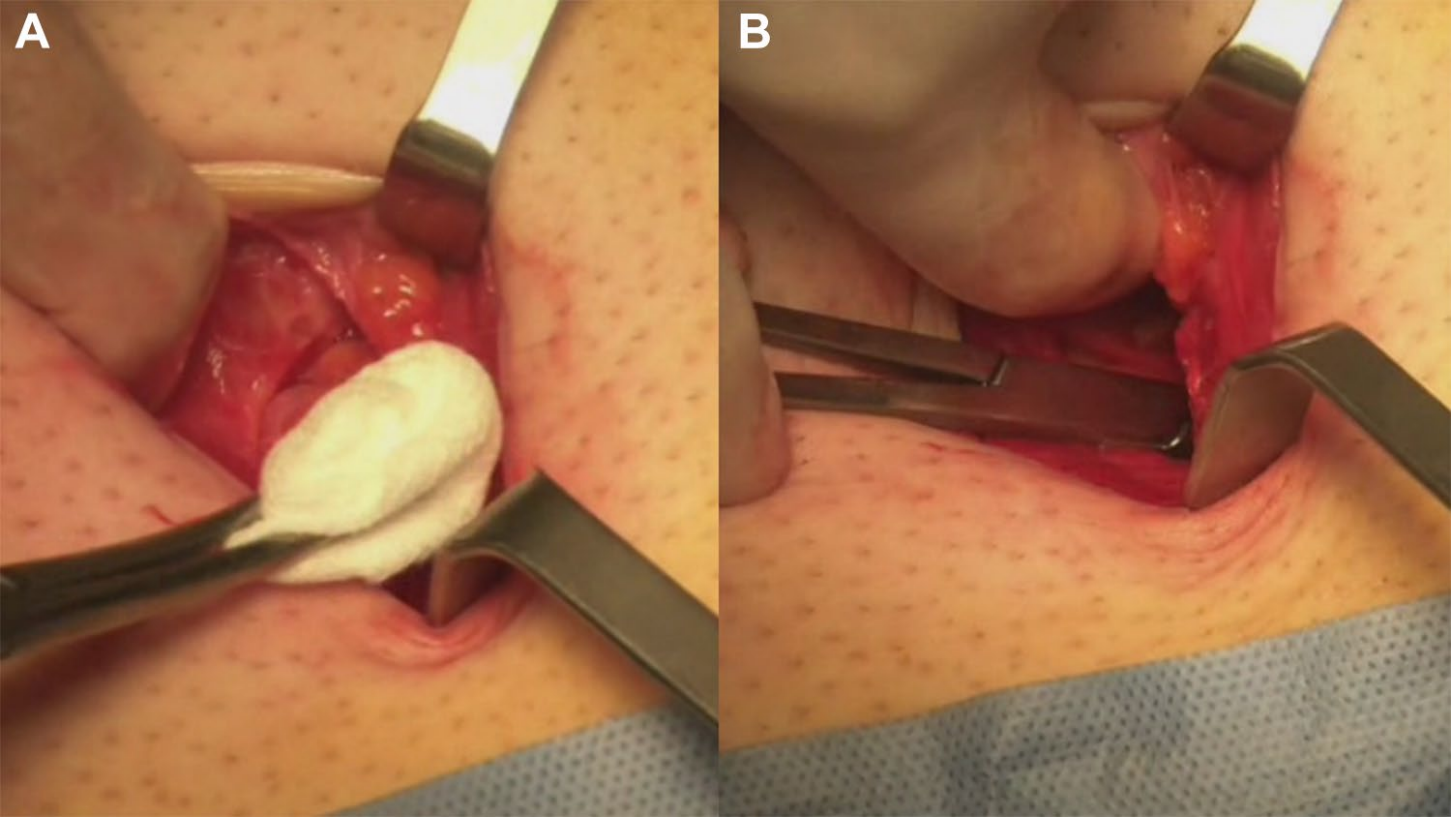
**a** Preparing the dissection of the peritoneal sheath from the posterior abdominal wall by means of a mounted pad. **b** The peritoneal dissection is made far away from hernia opening to allow the deployment of a large mesh for a broad overlap of the defect

**Fig. 5.**
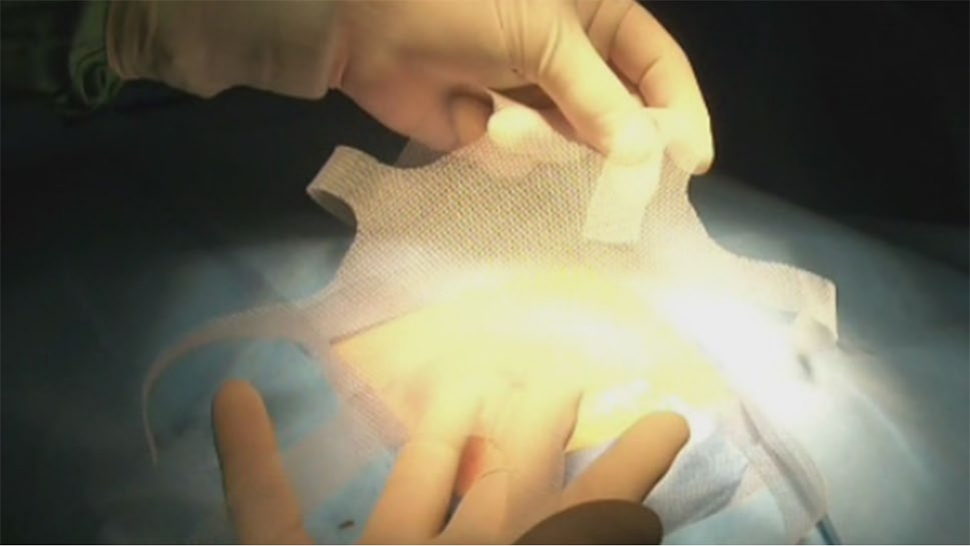
The tentacle shaped mesh before deployment in preperitoneal sublay

**Fig. 6.**
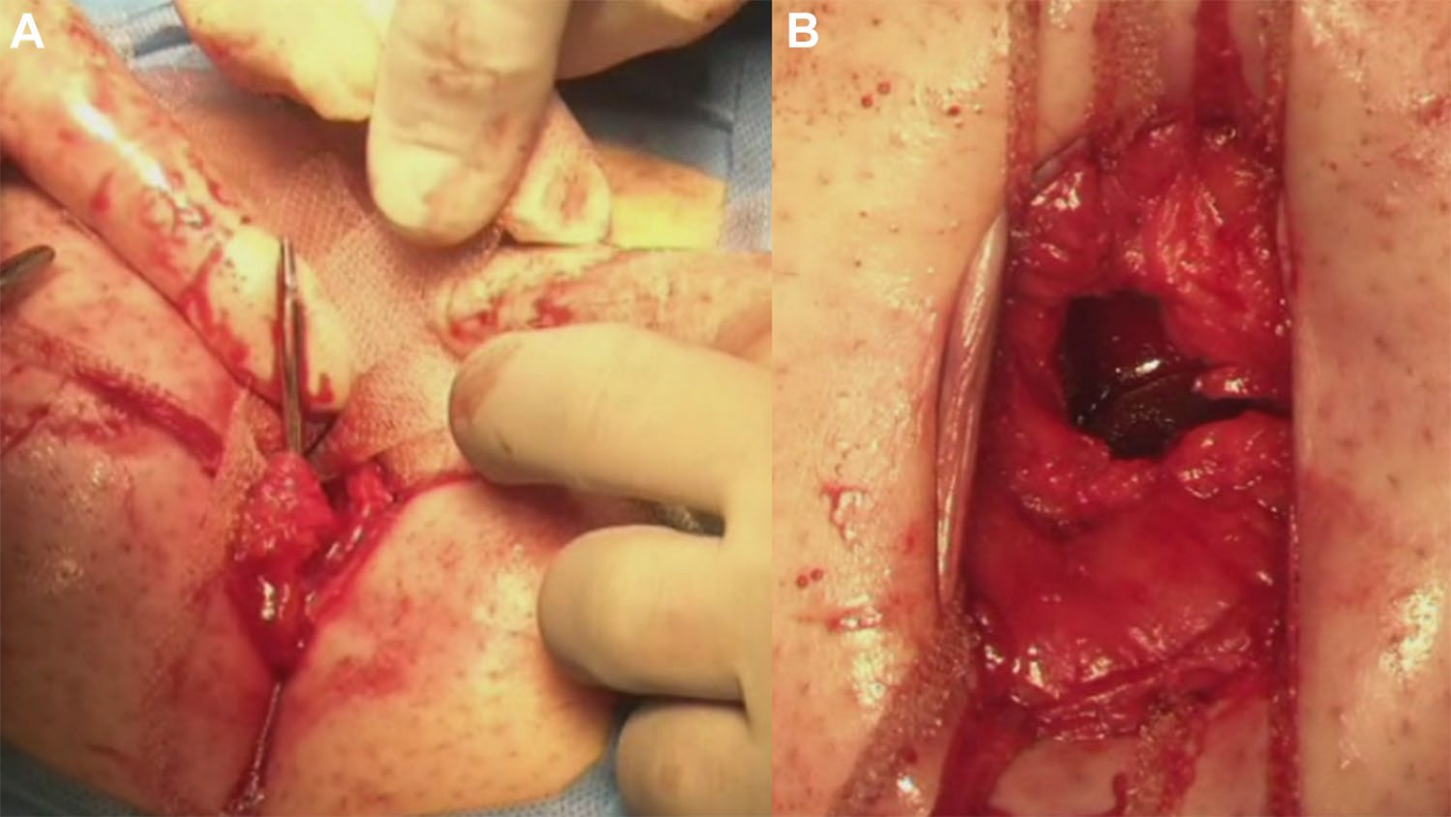
**a** The tentacles of the mesh are passed one by one through the abdominal wall with a proprietary passer. The strap edge is being inserted into the eye of the needle.** b** All 8 straps have been positioned crossing the abdominal wall far away from hernia defect. After pulling up the straps the flat mesh naturally deploys upon the peritoneal sheath assuring a wide coverage of the preperitoneal space

**Fig. 7.**
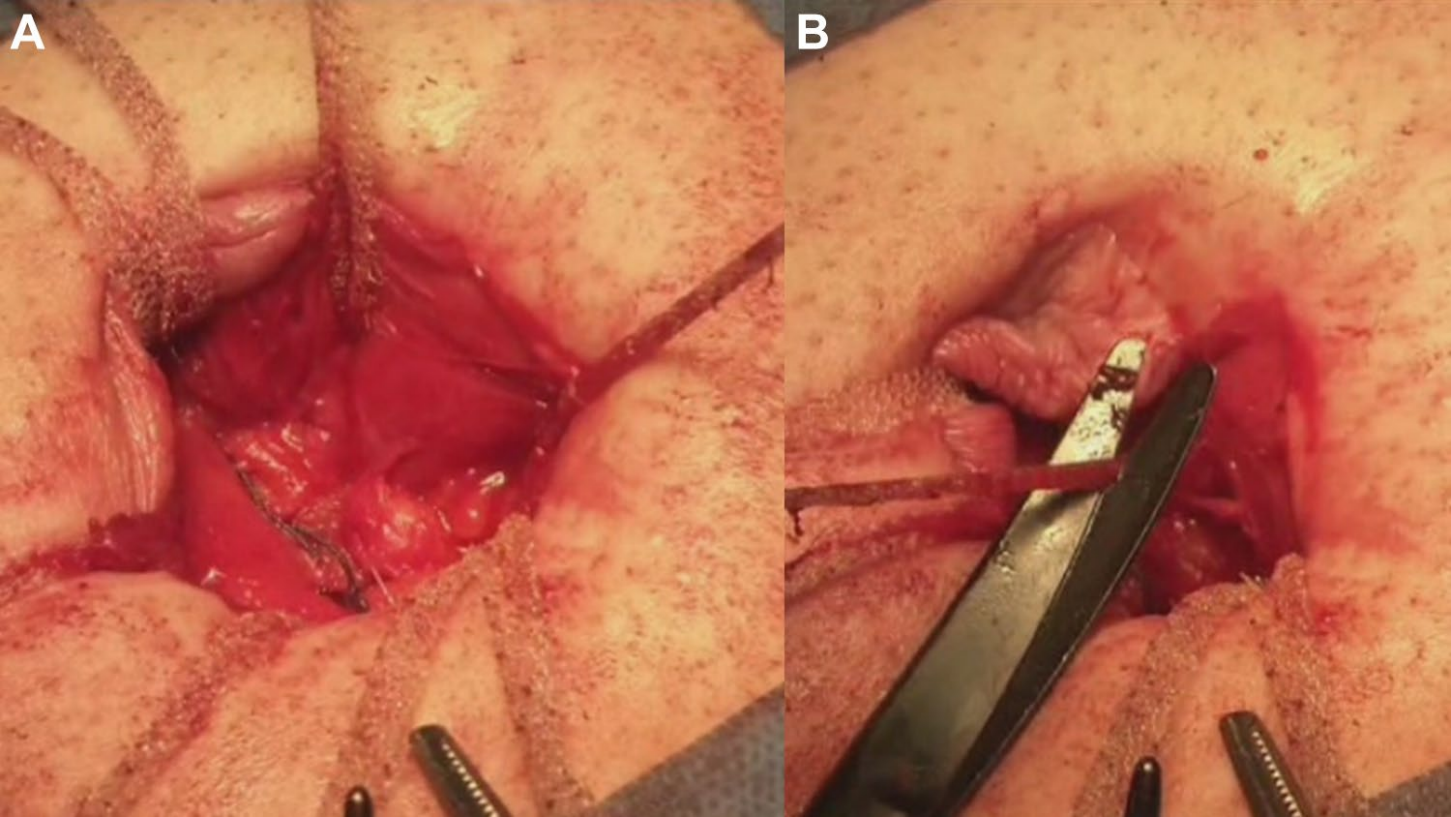
**a** The op site after closing the linea alba with running reasorbable suture. All 8 straps are exteriorized in the subcutaneous layer.** b** All straps are cut short one by one leaving a ca 2 cm stump in the subcutaneous layer

**Fig. 8.**
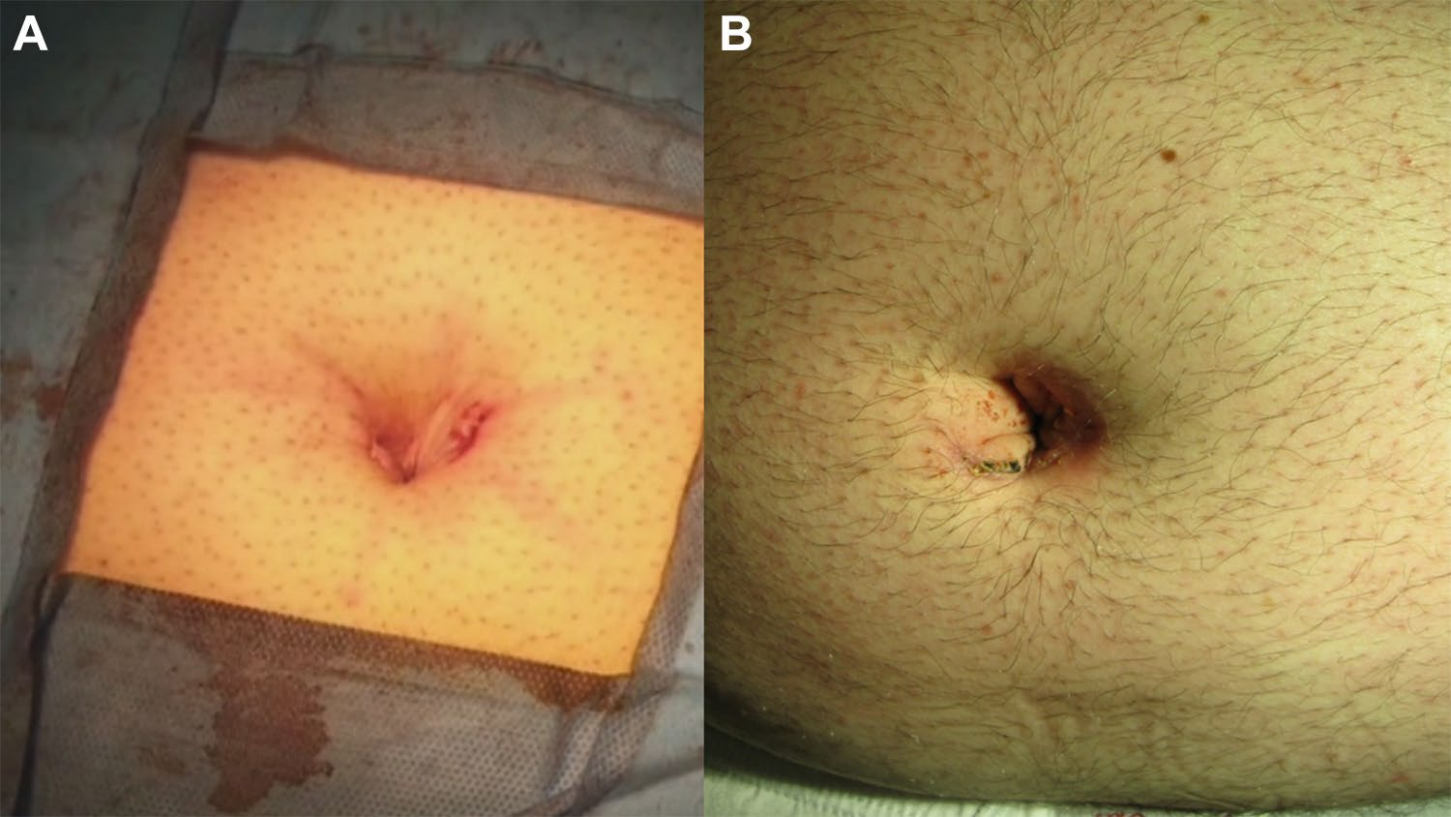
**a** The navel after reductive omphaloplasty and skin closure with total intrademal introverting stitches at the end of the procedure.** b** The umbilical scar one month post op

The institutional Ethics Committee approved this study. A cohort of 62 patients with hernia defect larger than 2 cm (medium and large defects according to the European Hernia Society classification) forms the body of this study. All patients, consecutively operated, underwent umbilical hernia repair with a specially designed implant composed of a central oval core and eight straps arising from the boundary edge at regular distances (Freedom Octomesh VHR XS produced by Insightra Medical Inc., USA). The body of the mesh used for the repair of navel hernias had an oval shape, sized 15 × 12 cm. The straps of the implant, positioned at the edge of the mesh, measure 2 cm in width and 15 cm in length. The straps are intended to be delivered laterally from the defect borders, from the preperitoneal space across the abdominal-wall structures holding the implant in place by friction. The implant is manufactured with lightweight, large-porous polypropylene mesh type 1, with a density of 70 g/m^2^ and pores > 10 µm. The procedure was performed in 62 patients (24 male and 38 female), with a mean age of 41 years (range 25–63), a median BMI of 29.40 (range 24–36). General anesthesia was administered in all individuals. A protocol-based antibiotic prophylaxis, as well as analgesic medication, were followed for 5 days postop. Forty-eight repaired umbilical hernias were primary protrusions, while 14 were recurrent (among these 3 multi-recurrent). Mean hernia defect size: 3.5 cm (range 2–5 cm). To carry out the procedure, a semilunar skin incision was made below the edge of the umbilical hollow (Fig. 1a, b). Once the hernia sac was dissected from the adhesion with the surrounding tissue, it was returned with its contents to the abdominal cavity (Figs. 2, 3a, b). Next, the peritoneal sheath was separated from the posterior rectus sheath with blunt dissection as far as possible from hernia opening creating a broad space for the placement of the implant (Fig. 4a, b). The tentacle mesh was then prepared to be delivered (Fig. 5). At this stage, thanks to a special needle passer specifically designed for this purpose, each strap could easily be deployed across the abdominal-wall tissue, entering the preperitoneal space distal from the hernia opening (Fig. 6a). In all 62 patients, the tentacle-shaped implant was placed in preperitoneal sublay below the posterior rectus sheath (Fig. 6b). Each implant was inserted to ensure adequate coverage and tension-free repair. After fascia closure, mesh tentacles were pulled high all together thus allowing spontaneous deployment of the mesh body above the peritoneum (Fig. 7a). Due to the variability of hernias repaired, duration of the mesh placement was determined starting from the return of the protrusion into the abdominal cavity and ending with the placement of the last tentacle. After closure of the linea alba, all 8 straps were trimmed leaving a ca 2 cm-long stump in the subcutaneous layer (Fig. 7b). In 42 of the operated patients, the redundant skin of the navel was excised to carry out an omphaloplasty through skin closure with total intradermal introverting stitches (Fig. 8a). This way, on completed wound healing, the navel could achieve an esthetically reasonable aspect (Fig. 8b). Postoperative follow-ups were at 1, 3, 6, and 12 months, and every subsequent year. The follow-up also included physical examination and real-time ultrasound (US) to look for implant and tentacle position. Further details concerning patient demographics, surgical treatment and related results are shown in Table 1.

**Table 1:** Patients demographics, treatment details, and results

Patient characteristics	Hernia types
Number of patients: 62	Primary umbilical hernia: 48 (16 incarcerated)
Gender: 24 male–38 female	Recurrent umbilical hernia: 14 (3 multi-recurrent– 5 incarcerated)
Mean age 41 years (25–63)	Mean hernia defect size: 3.5 cm ( 2–5 cm)
Mean BMI: 29.40 (24–36)	Total: 62

Procedure outcomes	Postoperative complications:
Mean mesh overlap: 6 cm (5–7 cm)	Wound infection: 0 (0%)
Time needed for mesh placement and strap positioning: mean 6 min (4–8 min)	Seroma: 4 (6%)^a^
Intraoperative complications: 0	Navel ischemia after omphaloplasty: 2 (3%)^a^
Hospital stay: 1 day (day surgery)	Recurrence: 0 (0%)
Mean follow-up length: 48 months (10–62 months)	Total complication rate: 15.4%

^a^Resolved within 15 days (conservative therapy)

## Results

In the cohort of 62 patients who underwent umbilical hernia repair with the tentacle-shaped implant, broad coverage of the abdominal wall far beyond the hernia opening was achieved. We measured a mean overlap of ca. 6 cm (range 5–7 cm) assessed from the hernia border to the lateral edge of the implant. The mean time needed to properly deliver all tentacles and deploy the mesh was 6 min (range 4–8 min). No suture fixation of the implant was necessary. The procedures were carried out in day surgery and the patients could be discharged on the same day of the operation. In the postoperative period, we observed 4 (6%) seromas and 2 ischemia of the navel skin after reconstruction. These minor complications were resolved within 15 days with conservative therapy. There were no wound infections, hematoma, chronic pain, or nerve-entrapment symptoms (Table 1). US scans during follow-up showed that all tentacles laid in the correct position. The placement of the tentacles would have unveiled an eventual implant dislocation, but no mesh dislocation was documented. During follow-up, no patient was lost. Patients were questioned regarding pain, any limitations to abdominal-wall mobility and overall satisfaction. In particular, no discomfort due to strap placement in the subcutaneous fat was reported. For all 62 patients, the mean follow-up was 48 months (range 10–62 months). No mesh dislocation or recurrence was reported. No significant pain or discomfort was described by the patients, even in the long term.

## Discussion

Umbilical hernia is commonly perceived as a disease requiring low attention from the surgical community. Despite this, many patients who undergo surgical repair of umbilical protrusion suffer from adverse events that could be avoided by improving the operative strategy using appropriate prosthetic devices. Among the complications that affect umbilical hernia repair, recurrence represents a controversial subject in literature. Usually, small defects (< 2 cm) are repaired open, with direct suture of the gap, while larger defects can be treated open or laparoscopic with the deployment of an implant in onlay or sublay fashion. Some studies involving large cohorts of patients indicate a recurrence rate of 1–10% after prosthetic umbilical hernia repair [8, 9]. Nevertheless, other reports specify an incidence of recurrences between 11 and 18% [4, 10]. Independent of these incongruences, in prosthetic umbilical repair, several factors are involved in the pathogenesis of recurrence: defect size, BMI, smoking, pregnancy, and reduced mesh overlap. A broad mesh coverage in a small surrounding like the umbilical area represents one of the most important problems that seems to be rarely debated in literature. Actually, the prosthetic coverage of a repaired umbilical defect is seldom larger than a few cm. This seems to be a major issue that, especially in obese patients with large defects, can facilitate the reappraisal of the protrusion. Another significant challenge is represented by mesh fixation at the boundary of the umbilical area, which is often extremely difficult and can lead to postoperative complications like tissue tear, hematoma and/or mesh dislodgment [11]. Furthermore, the position of the mesh used for reinforcing the abdominal wall also plays an important role. In the literature, it is well acknowledged that a deeper mesh placement helps in avoiding recurrences [12, 13]. For this reason, a preperitoneal sublay deployment of an implant seems to be more indicated to prevent recurrences. To overcome all these issues, a proprietary tentacle-shaped implant in different sizes has been developed for the surgical treatment of ventral and incisional hernia. The inherent friction force exerted by the tentacle straps crossing the abdominal-wall tissue has been already tested in an experimental trial on a porcine model to demonstrate the effectivity of the principle of mesh fixation through friction [14]. The tentacle mesh used to repair umbilical hernias in the above-described patient cohort seems to possess suitable characteristics to adequately resolve the problems connected to the surgical treatment of this frequent protrusion. Thanks to a well-established surgical strategy, achieving a broad dissection between posterior rectus sheath and peritoneal sheath does not appear challenging if carried out with careful separation of these elements. Once the preperitoneal planes are dissected, the delivery of the tentacle mesh by means of the proprietary passer is quick and easy; it allows the tentacles to pass from the posterior rectus sheath towards the subcutaneous layer crossing the abdominal wall. Piercing the abdominal wall with the passer, from the subcutaneous layer until the posterior rectus sheath, is quite easy. Actually, despite being carried out blind, the forefinger dome interposed between the posterior rectus sheath and the detached peritoneum consents a safe introduction and guidance of the passer needle through the preperitoneal interstitium as far as the wound opening. This maneuver also allows deployment of the mesh edges distal from the hernia border. Despite the distance from the hernia defect, there is no need for fixating the borders of the implant, the mesh is simply held in place by the friction exerted by the straps passing the abdominal wall structure laterally from the defect. The 3 mm-large passer needle tunneled across the tissue forces the 2 cm large strap to roll along its axis. Therefore, the friction exerted by its rolled contour into the small tunnel allows stable positioning of the arm within the tissues. This principle of physics has already been proven in the literature and is a suitable way to assure a fixation-free but firm positioning of polypropylene strips used for the treatment of female genital prolapse [15–19]. This kind of fixation-free implant stabilization is carried out under complete operator visualization, unlike point-to-point sutures carried out in a deep, narrow, and dark space. Delivery of the tentacle straps with the needle passer eliminates point fixation, thus simplifying the hernia repair procedure that can be performed in a shorter time. The limited mean time of 6 min needed for mesh placement and strap positioning seems to further confirm that the surgical repair can be carried out safely and quickly. The stabilizing feature of the tentacle strap system leads us to define its fixation-free effect as “freexation”. The surgical approach with the tentacle-shaped implant also revealed sharply reduced postoperative complications: the use of the tentacle mesh showed a reasonable postoperative complication rate of 9.7%. In the described patient sample, adverse events were represented only by 4 seromas, and 2 navel ischemia. The latter is a typical occurrence when, in large defects, the umbilical skin is tightly adherent to the hernia sack and after dissection remains with limited vascular support even if adequately resected before omphaloplasty. Thanks to the use of the tentacle-shaped mesh, among the patient sample, a mean mesh overlap from the hernia border ranging from 5 to 7 cm was assessed, which in this kind of procedure can be well defined as a broad overlap. It is the wide overlap assured by the tentacle mesh that is presumed to be the reason for the lack of recurrences in the examined patient cohort. In addition, postoperatively, all patients reported low pain that within 1 week sensibly diminished. Postoperative low pain seems to be linked to the non-fixation of the mesh to the myotendineal (and highly sensitive) surrounding of the umbilical region. In postoperative check-ups, easy localization of the straps with ultrasound scan was helpful, in particular in terms of recurrence prediction during patient follow-up which, to date, has not happened.

## Conclusions

Umbilical hernia repair with the described tentacle-shaped implant appears to yield improved results compared with conventional pure tissue or conventional prosthetic repair procedures. Aside from a quick, safe placement technique and the reduced rate of adverse events, usually reported in meshes that need fixation, a more significant benefit is determined by the very broad coverage assured by the implant shape and the related surgical technique. We deem it was the main reason why no recurrences were reported in the patients of the cohort. Overall, the advantages of using this proprietary designed implant are clearly demonstrated and result in fixation-free mesh placement, simplified procedure, broader coverage of the abdominal wall, shortening of operative time, and reduced intra- and early postoperative complications.
